# Effects of Nozzle Retraction Elimination on Spray Distribution in Middle-Posterior Turbinate Regions: A Comparative Study

**DOI:** 10.3390/pharmaceutics16050683

**Published:** 2024-05-19

**Authors:** Amr Seifelnasr, Xiuhua Si, Jinxiang Xi

**Affiliations:** 1 Department of Biomedical Engineering, University of Massachusetts, Lowell, MA 01854, USA; amr_seifelnasr@student.uml.edu; 2 Department of Aerospace, Industrial and Mechanical Engineering, California Baptist University, Riverside, CA 92504, USA; asi@calbaptist.edu

**Keywords:** nasal spray, actuation, particle size distribution, plume development, high-speed imaging, visualization, nasal valve, deposition distribution, liquid film translocation

## Abstract

The standard multi-dose nasal spray pump features an integrated actuator and nozzle, which inevitably causes a retraction of the nozzle tip during application. The retraction stroke is around 5.5 mm and drastically reduces the nozzle’s insertion depth, which further affects the initial nasal spray deposition and subsequent translocation, potentially increasing drug wastes and dosimetry variability. To address this issue, we designed a new spray pump that separated the nozzle from the actuator and connected them with a flexible tube, thereby eliminating nozzle retraction during application. The objective of this study is to test the new device’s performance in comparison to the conventional nasal pump in terms of spray generation, plume development, and dosimetry distribution. For both devices, the spray droplet size distribution was measured using a laser diffraction particle analyzer. Plume development was recorded with a high-definition camera. Nasal dosimetry was characterized in two transparent nasal cavity casts (normal and decongested) under two breathing conditions (breath-holding and constant inhalation). The nasal formulation was a 0.25% w/v methyl cellulose aqueous solution with a fluorescent dye. For each test case, the temporospatial spray translocation in the nasal cavity was recorded, and the final delivered doses were quantified in five nasal regions. The results indicate minor differences in droplet size distribution between the two devices. The nasal plume from the new device presents a narrower plume angle. The head orientation, the depth at which the nozzle is inserted into the nostril, and the administration angle play crucial roles in determining the initial deposition of nasal sprays as well as the subsequent translocation of the liquid film/droplets. Quantitative measurements of deposition distributions in the nasal models were augmented with visualization recordings to evaluate the delivery enhancements introduced by the new device. With an extension tube, the modified device produced a lower spray output and delivered lower doses in the front, middle, and back turbinate than the conventional nasal pump. However, sprays from the new device were observed to penetrate deeper into the nasal passages, predominantly through the middle-upper meatus. This resulted in consistently enhanced dosing in the middle-upper turbinate regions while at the cost of higher drug loss to the pharynx.

## 1. Introduction

Nasal spray pumps, celebrated for their non-invasive, efficient, and patient-compliant method of delivering a wide range of medications, are expanding beyond traditional uses like treating nasal allergies and the common cold to encompass a broader spectrum of therapeutic applications [1,2]. This includes serving as a practical approach for the topical management of conditions affecting the nose and paranasal sinuses, such as both forms of rhinitis and sinusitis [3,4,5]. Additionally, the nasal route is appealing for needle-free vaccinations and systemic medication delivery, which is especially valued for its rapid absorption and immediate effects [6]. These devices exemplify the shift toward intranasal administration as a compelling alternative to oral and injectable routes, with the nasal mucosa emerging as a promising conduit for systemic drug delivery [7,8]. The utility of nasal sprays in administering influenza vaccines underscores their potential in delivering treatments directly to areas rich in ACE2 receptors, such as the posterior two-thirds of the nose and nasopharynx, which is crucial for effective mucosal immunization against respiratory infectious pathogens like SARS-CoV-2 [9,10,11].

The effectiveness of nasal spray pumps in targeting specific areas within the nasal cavity is critically dependent on the intricacies of nasal cavity anatomy and functionality. These regions offer unique advantages for both targeted and systemic drug administration, which are influenced by factors including the drug formulation, the nasal spray pump design, the application method, and the deposition site, alongside the patient’s nasal health [12,13,14,15]. However, conventional nasal sprays often fail to deliver drugs effectively to the posterior turbinate region and nasopharynx due to the geometrical complexity of the nasal cavity. Most applied doses end up in the nasal vestibule and nasal valve, with only a fraction reaching the deeper turbinate. This limitation highlights the need for optimized spray properties and delivery methods to enhance dosimetry in the posterior nose [16,17,18,19,20].

Factors such as spray discharge velocity, aerosol mean diameter and distribution, spray plume angle, and release angle to the nostril play key roles in influencing intranasal dosimetry. Studies have shown that wider plumes and larger droplets tend to result in higher deposition in the anterior nose, while narrower plume angles and smaller droplets can improve delivery to the turbinate region. The orientation of spray release also affects deposition, emphasizing the importance of considering nasal physiology and the physical characteristics of the spray in achieving effective intranasal drug delivery [21,22,23]. Moreover, the success of medications administered through nasal spray pumps heavily relies on inspiratory flow. This flow is vital for moving the medication efficiently through the nasal pathways to the desired regions, ensuring effective drug delivery for optimal absorption or therapeutic impact [24]. The variability in individual nasal structures also plays an important role, markedly affecting medication distribution within the nasal passages and, consequently, the overall success of the intranasal delivery system [13].

Conventional nasal spray pumps are designed with an integrated actuator and nozzle that retract a distance equivalent to the piston’s stroke within the pump’s mechanism during application. A detailed examination of these devices reveals that the retraction stroke presents a design limitation, affecting the uniformity and precision of drug deposition. Effectively targeting the middle meatus, for instance, is recognized as a crucial strategy for the management of nasal polyposis and chronic rhinosinusitis [13,25,26]. The inconsistency introduced by nozzle retraction during the application process could undermine the delivery efficacy of protocols designed for conventional nasal spray pumps, which aim to deliver clinically significant medication doses to essential areas within the nasal cavity [14,27,28].

Recent advancements in nasal spray technology have led to the introduction of innovative devices that significantly enhance the delivery of medications to targeted areas within the nasal cavity, thereby optimizing therapeutic outcomes for a variety of conditions. Among these, devices such as Optinose’s XHANCE and Impel NeuroPharma’s INP104 are particularly noteworthy, each utilizing unique mechanisms to address the limitations observed in traditional nasal spray pumps, including the elimination of the nozzle retraction effect. XHANCE employs a breath-powered, bi-directional spray delivery system for the treatment of nasal polyps, using the action of a patient blowing into a mouthpiece to propel medication deeply into both nasal passages simultaneously. This mechanism ensures an effective distribution of medication to regions critical for absorption. By leveraging the patient’s breath through blowing, the device redirects the medication away from the nasal vestibule, thus achieving a more targeted and uniform distribution deep within the nose [6,29,30,31,32,33,34].

INP104, developed by Impel NeuroPharma, employs the Precision Olfactory Delivery (POD^®^) technology to administer a liquid formulation directly into the upper nasal cavity for acute migraine treatment. This specific delivery site is selected for its rich vascular network, ensuring rapid absorption into the bloodstream—crucial for swiftly managing migraine symptoms [35,36]. A distinctive feature of INP104 is its ability to overcome the limitations commonly associated with traditional nasal sprays, particularly the nozzle retraction issues. This is accomplished by integrating both the pumping mechanism and the actuator within the device’s body, thus effectively preventing nozzle retraction.

The objective of this study was to devise a modified standard multi-dose nasal spray pump based on a conventional design that eliminates nozzle retraction and utilize in vitro experimental methods to investigate its effect on the distribution of intranasal spray deposition, particularly within the middle and posterior turbinate regions. Specific aims include the following:

1. Design and develop a prototype of a modified standard multi-dose nasal spray pump with the nozzle separated from the actuator to eliminate nozzle retraction.
2. Utilize two different patient-specific partitioned transparent 3D-printed nasal cavity models to visualize the dosimetry and distribution of sprays administered intranasally from both the modified and conventional devices, using different head positions and inhalation flow conditions.
3. Quantify the mass of deposited spray droplets and film within the various sections of the nasal models (front nose section, including the nasal vestibule, turbinate regions, and the nasopharynx).
4. Perform a comparative analysis of the experimental results to assess the dosimetry distribution between the conventional and modified devices, specifically evaluating the enhanced effectiveness of the modified device in delivering nasal sprays, focusing on the middle and posterior turbinate regions.

## 2. Materials and Methods

### 2.1. Study Design

**Figure 1:**
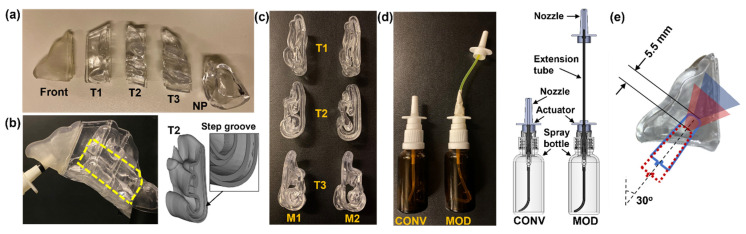
Nasal models and devices: (**a**) Sectional casts for regional deposition quantification (front nose, turbinate 1 [T1], turbinate 2 [T2], turbinate 3 [T3], and nasopharynx [NP]); (**b**) Target region (middle and posterior turbinate) outlined in dashed yellow lines; (**c**) Cross-sectional views of the sectional casts of the two nasal models (M1 and M2); (**d**) A conventional nasal spray pump (CONV) and a modified nasal spray pump with the nozzle attached via a flexible tube to the pumping mechanism (MOD); (**e**) Illustration of the nozzle position and spray plumes with and without retraction (red vs. blue).

This study aimed to investigate the distribution of intranasal spray deposition between two types of nasal spray pumps (Figure 1): a conventional multi-dose nasal spray pump (Hengni), hereafter referred to as CONV, and a modified version (MOD).

The MOD device was adapted from a conventional multi-dose nasal spray pump by detaching the nozzle from the actuator and connecting it to the dispensing mechanism with a flexible plastic tube, thereby eliminating nozzle retraction during application. The experimental setup utilized two multi-piece, transparent, and anatomically accurate models of the left nasal passage of a healthy adult male (Figure 1a–c), which were previously used in a study assessing the natural nasal cycle’s effect on intranasal spray deposition [24]. These models, named N1 (negative 1) and P1 (positive 1) in the previous study [24] and renamed model 1 (M1) and model 2 (M2) in this study, feature distinct turbinate geometries to represent variations in nasal passage morphologies. Each cast consisted of five sections—front, turbinate 1 (T1), turbinate 2 (T2), turbinate 3 (T3), and nasopharynx (NP)—with step-shaped grooves at the interface for tight seal (Figure 1). The formulation used in both CONV and MOD devices, a slightly viscous 0.25% w/v methyl cellulose (MC) aqueous solution, was specifically chosen to enhance the adherence of deposited droplets and liquid film within the nasal cavity models. This choice aimed to prevent the undesired movement of liquid between deposition spots during disassembly, which is crucial for the accurate quantification of local deposition measurements.

To ensure consistency, each device’s nozzle was positioned at a 30° angle from the nostril’s normal and inserted to a depth of 10 mm for all tests. A single application was administered per test to quantify and analyze spray deposition under varying conditions. Moreover, the study design incorporated two head orientations (22.5° and 45° backward tilts) to leverage gravitational effects, facilitating spray deposition within the middle and posterior turbinate regions. Several previous studies have shown that backward head tilts could enhance deposition in the middle and posterior turbinate regions [37,38]. Additionally, two inhalation airflow scenarios were simulated—one representing a breath-hold (i.e., no flow) and the other a constant inhalation at 16 L/min—using a flow meter (Omega, FL-510, Stamford, CT, USA) for regulation. The flow rate of 16 L/min was chosen to simulate a gentle inhalation during spray administration [39].

For the experimental setup, the nasal models (Figure 1) were assembled with clear adhesive tape securing the sections (Front, T1, T2, T3, and NP) and positioned to match the specified head orientations. After being filled with the MC solution and adequately primed, the nasal spray pumps were positioned accordingly. Clamps with articulating arms were used to fix the nasal models and both the CONV and MOD nasal spray pumps in their respective positions. However, compared to the CONV device, an additional clamp was used to fix the MOD device’s nozzle in place within the nasal models’ vestibule at the set administration angle and insertion depth. A vacuum (Robinair 3 CFM, Warren, MI) was used to simulate a constant inhalation through the NP during tests that involved airflow. Visualization of spray dynamics and deposition patterns was facilitated by a fluorescent green dye (GLO Effex, Murrieta, CA, USA), captured using a high-definition camera (Canon EOS Rebel T7). The use of LED (light-emitting diode) lighting within the 385 to 395 nm wavelength range highlighted the dye, thereby accentuating the spray particles, deposited droplets, and liquid film for enhanced observation. The mass of deposited liquid in each nasal section was quantified before and after tests using a precision electronic scale (Bonvoisin, San Jose, CA, USA), ensuring a minimum of five repetitions for each condition to address variability.

Through this approach, the study aimed to compare the dosimetry distribution between CONV and MOD, employing both quantitative and visual assessments with special emphasis on the enhanced effectiveness of the MOD device in delivering the spray formulation effectively to the upper, middle, and posterior turbinate regions.

### 2.2. Modified Nasal Spray Pump (MOD)

In the study, a standard multi-dose nasal spray pump was modified to allow the nozzle to be fixed in position during spray application, thus eliminating nozzle retraction and aiming to improve drug deposition in specific nasal regions (MOD, Figure 1d). The nozzle of the standard pump is integrated with the finger-actuated applicator into one single plastic component, which is mounted onto the rigid output tube of the device’s pumping mechanism. For the MOD device, the integrated nozzle was sheared off at its base, exposing the central channel through which pressurized liquid dispensed via the pumping mechanism flows during device application prior to reaching the nozzle tip. A narrow, elongated, flexible tube was inserted between the base of a separate nozzle component and the end of the exposed output channel of the modified applicator component. This arrangement enabled the dispensed pressurized liquid to flow to the nozzle, which was fixed in place within the nasal model’s vestibule during the experimental runs. Dow Corning 732 clear sealant (Dow Corning, Midland, MD, USA) was used to secure the elongated flexible tube in place. This tube had the same inner and outer diameters as the dip tube placed in the nasal spray bottle and was made from the same material, ensuring that the frictional characteristics of the flow path remained consistent with the original design. The extension tube was 100 mm in length, with an inner diameter of 1.2 mm and an outer diameter of 2.1 mm. The extension tube length was set at 100 mm to provide just enough slack during spray application, preventing nozzle retraction. This modification enabled the creation of slack in the tube when the device was secured in place during the conduction of the experimental runs. The slack in the tube prevented retraction of the nozzle during spray application by accommodating the separate applicator’s movement at the base of the flexible extension tube, with the nozzle fixed at the predetermined insertion depth within the nasal model’s vestibule, thereby eliminating backward movement of the nozzle. This design was hypothesized to enhance the targeting of medication to the middle and posterior nose. To ensure testing consistency, the same force and speed were applied to the MOD and CONV devices during spray actuations.

### 2.3. Nasal Cavity Models

The nasal models, M1 and M2 (Figure 1), were developed from MRI scans of a 53-year-old male [14,24]. Both models were created based on the left nasal passage, including the nasal septum, to allow for clear visualization of spray delivery, liquid film mobilization, and final deposition distribution within the turbulent region during the experimental runs. This visualization was achievable not only from the lateral side but also from the relatively flatter septum side. The turbulent regions of both M1 and M2 were modified by altering the thickness of their conchae, leading to distinct nasal passage morphologies between M1, with a more congested pathway, and M2. These changes were implemented to introduce intersubject variability into the study, focusing on the variations in nasal passage morphology among subjects. The cross-sectional views of the models, illustrating these differences in turbinate thickness, are shown in Figure 1c. The geometric modifications were achieved through the use of SolidWorks (Dassault Systems, Waltham, MA, USA).

A Formlabs 3D printer (Form 3B+, Somerville, MA, USA) was used to create the nasal casts, employing a clear, rigid stereolithography (SLA) resin (Formlabs Clear Resin, FLGPCL04), which resulted in transparent models (Figure 1). The ‘front’ section of the nose was crafted using an elastic SLA resin (Formlabs Elastic 50A Resin, FLELCL01) with the same printer, chosen for its ability to mimic the flexibility of this particular section. This allowed for the dilation necessary for deeper insertion of the nasal spray pump’s nozzle to reach the desired depth. Employing transparent casts offers the significant advantage of enabling the visual tracking of liquid spray dynamics and film formation within the model in real time.

### 2.4. Spray Characterization

To compare the CONV and MOD devices and to understand the design modifications’ effects, a thorough analysis of the spray characteristics of the solution used in this study (0.25% MC aqueous solution), which was dispensed from both devices, was essential. Accordingly, a laser diffraction spray particle size analyzer (Spraylink, Dickinson, TX, USA) was used to precisely measure the size distribution of spray particles per application dispensed from both the CONV and MOD devices. To account for variability in spray characteristics during application, which was due to application force and speed, multiple measurements were taken, and the results were averaged. Care was taken to dispense the sprays from both devices in an equally forceful and swift manner, thus minimizing variability during spray application. Furthermore, the mass per spray dose dispensed from both devices was measured using a precision electronic scale (Bonvoisin, San Jose, CA, USA), calculated as the difference in device mass before and after spray application. To mitigate variability, several measurements were conducted, and the results were averaged.

**Figure 2:**
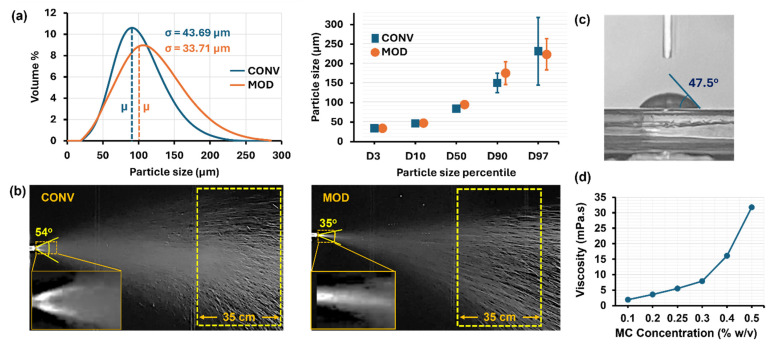
Characteristics of the 0.25% w/v methylcellulose liquid spray dispensed from both the conventional (CONV) and modified (MOD) devices following a single dose application: (**a**) Particle size distribution, with mean (μ) and standard deviation (σ) indicated; (**b**) Images of the liquid spray plume generated by each device; (**c**) Contact angle of a drop of 0.25% w/v MC aqueous solution on 3D-printed rigid transparent SLA resin; (**d**) Dynamic viscosity as a function of MC concentration (% w/v) in an aqueous solution.

Furthermore, considering the important roles of spray plume angle, morphology, and dispersion in determining delivery efficiency, the plumes dispensed from both the CONV and MOD devices were captured using a Canon EOS Rebel T7 camera. This enabled detailed comparisons of the spray plumes, providing insights into how design modifications influenced the plume angle, dispersion, and, consequently, the delivery efficiency of the nasal spray. Capturing the plume allowed for an in-depth examination of its dynamics, enabling a thorough evaluation of the impact of the MOD device. The contact angle of the MC solution on an SLA resin plate was measured to be 47.5° (Figure 1c), and the solution viscosity was measured to be 5.5 mPaˑs (i.e., at 0.25% w/v concentration, Figure 2d).

### 2.5. Statistical Analysis

Statistical evaluations were carried out using Minitab software 22 (State College, PA, USA). Variability in the results was examined through a one-way analysis of variance (ANOVA). The deposited masses in each experimental scenario were represented and described as mean ± standard deviation. To visualize the deposition data statistics, violin plots were utilized; these plots resemble box plots but also show the probability density of the data across different values, which is smoothed out by a kernel density estimator [40].

## 3. Results

### 3.1. Spray Characteristic Comparisons between CONV and MOD Devices

#### 3.1.1. Particle Size Distribution

From the particle size distribution curves depicted in the left panel of Figure 2a, it was evident that the mean particle size associated with the modified device was greater than that of the conventional nasal spray pump (i.e., 101.704 μm vs. 90.456 μm, respectively). Additionally, the distribution curve of the modified device exhibited a larger variance, indicating that not only were the average particle sizes larger, but the spread of sizes was also wider, suggesting a general trend toward larger particle sizes being generated by the modified device compared to the conventional one. Furthermore, both curves exhibited a right-skewed distribution, reinforcing the presence of larger particles. Moreover, the median particle diameter (D50, shown in Figure 2a, right panel) of the spray dispensed from MOD was only slightly greater than that from CONV (93.64 μm vs. 84.48 μm, respectively).

#### 3.1.2. Plume Geometry

Figure 2b illustrates the spray plumes dispensed from the two devices, CONV and MOD. The MOD device generated a spray plume with a narrower angle than that of CONV (35° versus 54°, respectively). Further examination of the spray plume geometries dispensed by both the CONV and MOD devices revealed that the plume produced by the MOD device displayed a higher density of visible streaks further downstream, as indicated within the region enclosed by the yellow dashed rectangle. This distinction suggests a variance in droplet size between the two plumes, with the increased streak density in the MOD device’s plume implying the presence of larger droplets within this region. These larger droplets tend to scatter more light, making them more visible in imagery. This qualitative observation aligns with quantitative data from laser diffraction particle size analyzer measurements, which confirmed a higher density of larger droplets in the spray plume from the MOD device compared to that from the CONV device. The narrower plume angle of MOD could be attributed to the lower exit velocity resulting from its integrated extension tube. Despite this, variations in the range of the spray plume—the measure of its furthest reach—were not noticeably different between devices.

#### 3.1.3. Mass per Dose Comparison: CONV vs. MOD Devices

Based on ten measurements with a precision mass scale, the average mass of the 0.25% aqueous solution dispensed per spray application from the CONV device was recorded as 160.3 mg. For the MOD device, and with the same number of measurements, the average mass dispensed per spray application was 143.2 mg.

### 3.2. Spray Deposition Distribution vs. Device Type and Head Angle

**Figure 3:**
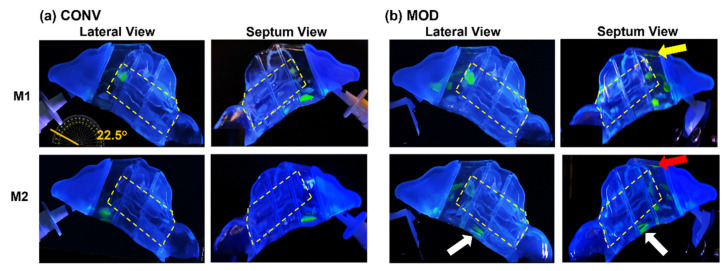
Liquid deposition within nasal models M1 and M2 at a 22.5° backward head tilt during a breath-hold (i.e., no flow), captured from the lateral and septum sides of each model after one dose of spray application from both devices: (**a**) CONV; (**b**) MOD. The target region is outlined in dashed yellow lines. Different arrow colors were used solely to distinguish different regions of interest.

Figure 3 visualizes the spray distribution across various nasal cavity regions (i.e., front nose, T1, T2, T3, and NP) in the M1 and M2 models. Two important factors should be considered when interpreting the deposition in this figure and hereafter. Firstly, as previously mentioned, the average mass per spray dosage dispensed per application was higher for CONV than for MOD, at 160.3 mg versus 143.2 mg, respectively. In other words, MOD delivered around 11% less dosage on average compared to CONV. Secondly, it is important to note that the mass deposition measurements within the turbinate region sections of the nasal models (T1, T2, and T3) represent the total depositions within these areas. The nature of the models precluded separate measurements of deposition distributions across the heights of these sections to quantify the deposited mass in the upper, middle, and inferior parts of the turbinate regions. Therefore, to accurately assess the effectiveness of spray delivery to the targeted regions of interest (i.e., middle and posterior turbinate regions) with each device (CONV and MOD), it was necessary to complement the section-specific deposition mass measurements with visualization recording results.

#### 3.2.1. Spray Deposition vs. Device at a 22.5° Backward Head Tilt (Breath-Hold)

At a 22.5° back-tilted head position, a single-dose application using the nasal spray pump with the conventional retracting nozzle (CONV) and no inhalation airflow (mimicking a breath-hold scenario) resulted in the dispersion of liquid droplets and film within the middle and inferior regions of the T1 turbinate section. In the M1 model, following a one-dose application, the liquid film primarily settled within the anterior middle turbinate and the floor of T1 (Figure 3a, upper two panels). The less congested M2 model (Figure 3a, lower two panels), featuring wider inferior passageways (airspace), showed the final settling of liquid film across the floor of T1, as observed from both lateral and septum views.

When using the nasal spray pump with the modified nozzle (MOD) that remained fixed during application, the resulting liquid behavior and deposition pattern were notably altered, as shown in Figure 3b. In the M1 model (upper two panels), there was enhanced coverage of the middle and upper-middle sections of T1, with a marginally greater deposition in the anterior middle meatus and reduced film deposition in the inferior T1. A small amount of film deposition was also noted along the roof of T1 (indicated by a yellow arrow). The elimination of the retraction effect during spray administration led to a narrower spray plume cross-section near the nasal valve area (as demonstrated in the schematic presented in Figure 1e), resulting in deeper and more superior penetration of the spray, contributing to the observed differences in deposition patterns. In contrast, the M2 model (Figure 3b, lower two panels) displayed a more dispersed liquid droplet/film dosimetry pattern in the middle T1 regions, with some minor deposition extending superiorly across the roof (indicated by a red arrow). The M2 model’s wider inferior passageways also aided in the translocation and deposition of the liquid film in the inferior T2 section (indicated by a white arrow).

**Figure 4:**
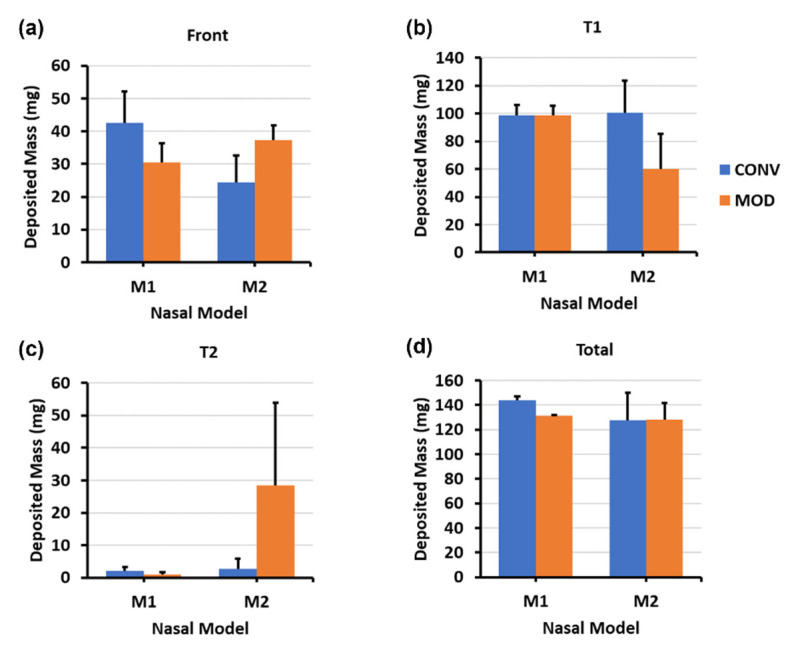
Deposition variation vs. nozzle type (retracting CONV and fixed MOD) in different regions of the nose with a 22.5° back tilt head position and during a breath-hold for nasal models M1 and M2: (**a**) Front nose; (**b**) T1; (**c**) T2; (**d**) Total deposition.

Figure 4 depicts the mass deposition variations. The mean mass deposition in the T1 section was almost equal when both devices (CONV and MOD) were used to administer sprays in the M1 model (Figure 4b), whereas it was less in the M2 model (100.45 mg for CONV and 59.86 mg for MOD). However, using the MOD device resulted in a larger deposition in T2 than when CONV was used in M2 (28.32 mg vs. 2.75 mg, respectively). The deposition in T2 of the M1 model was low using both devices. Complementing the quantification results with the visualizations, most of the mass deposited in the T1 section via MOD pertained to liquid droplets and film that were distributed within the upper and middle sections of M1 and M2, including the anterior middle meatus, as opposed to mostly deposition along the floor with the CONV device. Moreover, in the M1 model, on average, there was lower deposition in the front nasal region, including the nasal vestibule, using the modified (MOD) device compared to the conventional (CONV) device (30.43 mg vs. 42.63 mg, respectively. However, the opposite was observed in the M2 model, where deposition in the front nose section was greater using MOD than CONV (37.38 mg vs. 24.48 mg, respectively). No deposition was observed in either the T3 or the nasopharynx when either device was used to administer spray in the nasal models.

#### 3.2.2. Spray Deposition vs. Device at a 45° Backward Head Tilt (Breath-Hold)

**Figure 5:**
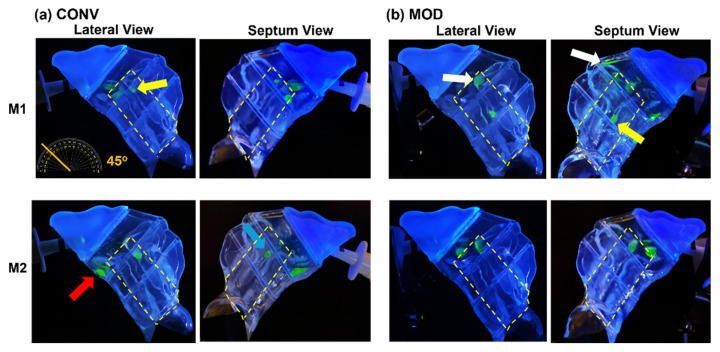
Liquid deposition within nasal models M1 and M2 at a 45° backward head tilt during a breath-hold, captured from the lateral and septum sides of each model after one dose of spray application from both devices: (a) CONV, and (b) MOD. The target region is outlined in dashed yellow lines. The arrows highlight major differences in deposition.

Figure 5 visualizes the deposition distributions at a 45° backward head tilt with a single-dose application from the nasal spray pump. With the conventional retracting nozzle (CONV) in the M1 model, using no inhalation airflow (i.e., a breath-hold), the deposition is confined to the T1 turbinate region (Figure 5a, upper two panels). The increased head tilt angle led to the liquid film predominantly settling in the middle region of T1, with some slight deposition in the anterior middle meatus (yellow arrow) and no deposition in the inferior region or on the floor. 

This outcome may be attributed to the interplay between the methylcellulose (MC) solution’s surface tension, adhesion properties, holding capacity of the liquid, and the effects of gravity at the points of deposition. The formation of larger droplets or a wall film in these areas might increase the risk of film breakup, prompting subsequent translocation either downward or posteriorly into adjacent turbinate sections driven by destabilizing gravitational forces. Conversely, spray administered in the M2 model via CONV resulted in mostly inferior deposition within the T1 section that finally settled along the floor of the same section (red arrow, Figure 5a, lower two panels). Lesser liquid deposition was also observed in the middle meatus in the frontward segment of the T2 section (orange arrow).

In contrast, using the MOD device in the M1 model led to greater deposition initially in the upper middle and superior sections of T1, including the anterior middle meatus (white arrows, Figure 5b, upper two panels), with some minor liquid film deposition on the floor of T1. The larger gravitational force component along the middle turbinate region, which is due to the larger head angle, facilitated the breakup of the resulting liquid film that formed and deposited in the anterior middle meatus and led to subsequent liquid film translocation initially along the middle meatus. The film was then dragged diagonally and slightly inferiorly until it settled on the upper outer surface of the inferior turbinate in the T2 region (yellow arrow). It was also observed that spray administered via MOD in M2 (Figure 5b, lower two panels) resulted in mostly deposition in the middle section of T1, including in the anterior middle meatus, as opposed to the mostly inferior liquid film deposition in the same model when CONV was used.

**Figure 6:**
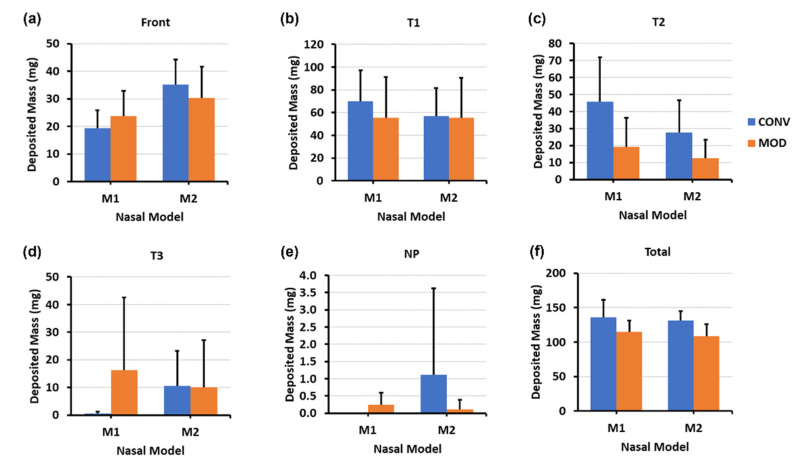
Deposition variation vs. nozzle type (retracting CONV and fixed MOD) in different regions of the nose with a 45° back tilt head position and during a breath-hold for nasal models M1 and M2: (**a**) front nose, (**b**) T1, (**c**) T2, (**d**) T3, (**e**) nasopharynx (NP), and (**f**) total deposition.

The mass deposition distributions within the various model sections using both devices are portrayed in Figure 6. The differences in mean mass depositions in the T1 section (Figure 6b) were small between both devices in the M2 model (57.10 mg for CONV and 55.62 mg for MOD). The difference was slightly higher in M1 (70.20 mg for CONV and 55.42 mg for MOD). However, as mentioned above, from the visualization captures, the deposition distribution in the T1 section using CONV was either around the lower middle region or on the floor of T1, as opposed to the deposition pattern pertaining to MOD, which was generally within the upper middle and/or superior regions. 

Compared to the 22.5° Backward Head Tilt, at 45°, there was enhanced mass deposition in both T2 and T3 as well as some very marginal deposition within NP (Figure 6c–e). Although the mean deposited mass values in T2 appear to be larger for CONV compared to MOD, it is important to note that deposition that occurred in T2 and T3 using CONV was mostly along the floor of the nasal cavity as opposed to the case with MOD, where deposition in T2 and T3 was within the middle and lower middle regions. In the front nose section, the mean mass of deposited liquid was lower for MOD than CONV in M2 (30.26 mg vs. 35.16 mg, respectively) and was slightly larger for MOD than CONV in M1 (23.68 mg vs. 19.34 mg, respectively).

#### 3.2.3. Spray Deposition vs. Device at a 22.5° Backward Head Tilt (Constant Inhalation)

**Figure 7:**
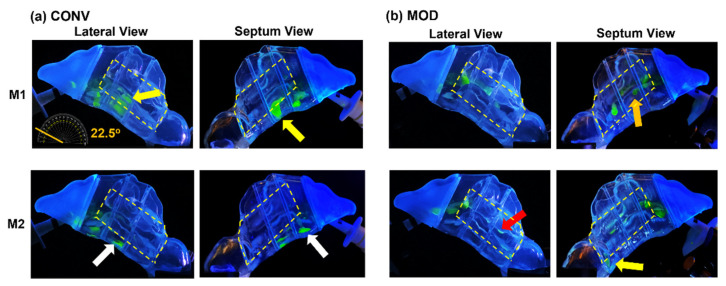
Liquid deposition within nasal models M1 and M2 at a 22.5° backward head tilt and a constant inhalation airflow (16 L/min), captured from the lateral and septum sides of each model after one dose of spray application from both devices: (**a**) CONV, and (**b**) MOD. The target region is outlined in dashed yellow lines.

Under the effect of a constant inhalation flow of 16 L/min and a head orientation of 22.5° titled backward, the retraction of the nozzle of the CONV device during spray application resulted in deposition within the inferior turbinate region within the T1 and T2 sections in both the M1 and M2 models (Figure 7a). Because M1 was more congested than M2, its narrower passageways within the vicinity of the inferior turbinate and between its outer lining and that of the nasal septum resulted in drastically different liquid film behavior. Spray application within M1 resulted in the scattering of liquid droplets and film across the lower half of T1, covering the lateral wall, anterior septum, and inferior turbinate. Flow shear resulting from airflow across the inferior turbinate (along with gravitational effects) dragged liquid film, which was initially deposited within the middle T1 section into T2, filling and spreading across the narrow cavity in T2 between the septum and the inferior turbinate, covering a large portion of the inferior turbinate’s outer surface (yellow arrow, Figure 7a, upper panels). On the other hand, compared to M1, when liquid spray was dispensed within the M2 model using CONV, the wider spaces within the inferior meatus and between the inferior turbinate, the adjacent lateral wall, and the septum, resulted in a more scattered deposition of smaller droplets and film within both the T1 and T2 turbinate sections, with liquid droplets finally settling mostly on the floor of T1 and T2 (white arrow, Figure 7a, lower panels). Deeper deposition into T2 was due to the liquid film translocation along the floor of the nasal cavity due to flow shear and gravity.

In contrast, the fixed nozzle of the MOD device at the set administration angle and insertion depth led to liquid delivery directly to the middle turbinate region and middle meatus in both the M1 and M2 models, with minor deposition within the nasal vestibule and the front nose section. Both models also exhibited liquid film development and deposition across the middle section of T1, leading toward the middle turbinate and middle meatus. Flow shear from the dominant airflow through the middle meatus facilitated the dragging of the liquid film along the entire middle meatus and toward the nasal cavity’s posterior region via flow shear within the T1, T2, and T3 sections. The effects of gravity were twofold: the smaller component along the sloped middle meatus assisted in facilitating translocation along the middle passageway; however, the larger component normal to the middle meatus swayed liquid droplets and film out of the middle meatus, making them drip and settle either along the lower edge of the middle turbinate within T2 and T3 (red arrow) or on the upper outer surface of the inferior turbinate (orange arrow). The narrow space between the outer surface of the inferior turbinate and the nasal septum of the M1 model resulted in the final settling of the liquid film in the posterior cavity region (i.e., T3) between the upper outer surface of the inferior turbinate and the septum. However, the wider spaces in the M2 model within the caudal nasal cavity region resulted in the scattering of the liquid droplets and film that descended from the middle meatus across the lower region of the posterior inferior turbinate, with some slight deposition at the entrance to the nasopharynx (yellow arrow, Figure 7b, lower right panel).

**Figure 8:**
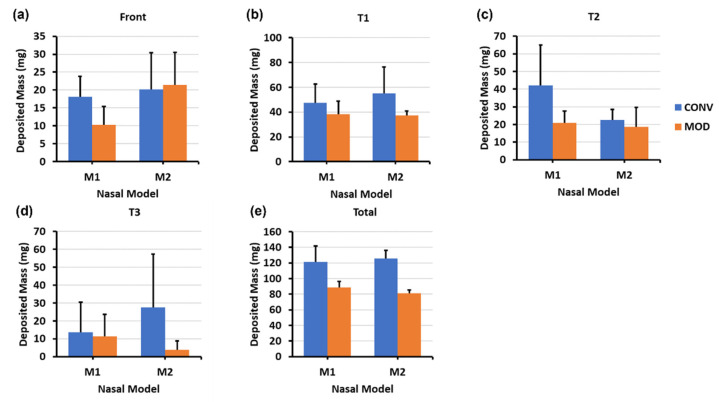
Deposition variation vs. nozzle type (retracting CONV and fixed MOD) in different regions of the nose with a 22.5° back tilt head position and a constant 16 L/min inhalation airflow for nasal models M1 and M2: (**a**) front nose, (**b**) T1, (**c**) T2, (**d**) T3, and (**e**) total deposition.

As shown in the clustered column charts associated with the mass deposition distributions (Figure 8), utilizing the delivery protocol at the 22.5° backward tilted head angle with the aid of constant inhalation airflow notably enhanced the quantitative deposition distribution within both models using both devices, particularly within the T2 and T3 sections. However, even though the deposited mass within the various turbinate sections using CONV in both M1 and M2 was generally higher than MOD (for example, 22.54 mg vs. 18.60, respectively, in T2 of M2), deposition with CONV was distributed across the lower half of the turbinate sections and along the nasal floor. In contrast, the deposition distribution with MOD targeted the middle and posterior cavities and was statistically consistent in both the M1 and M2 models. Additionally, the mean deposited mass in the front nose section of M1 was lower for MOD compared to CONV (10.22 mg vs. 18.06 mg, respectively). Front nose mass deposition was close between CONV and MOD in M2.

#### 3.2.4. Spray Deposition vs. Device at a 45° Backward Head Tilt (Constant Inhalation)

**Figure 9:**
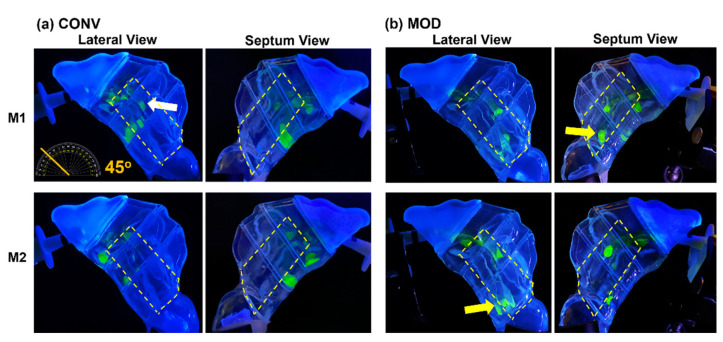
Liquid deposition within nasal models M1 and M2 at a 45° backward head tilt and a constant inhalation airflow (16 L/min), captured from the lateral and septum sides of each model after one dose of spray application from both devices: (**a**) CONV, and (**b**) MOD. The target region is outlined in dashed yellow lines.

At a 45° backward head orientation and using a 16 L/min constant inhalation airflow, the deposition pattern in both M1 and M2 using both the CONV and MOD devices followed the same trend as observed at a 22.5° backward head tilt under the same inhalation flow rate of 16 L/min (Figure 9). However, the steeper angle resulted in greater gravitational effects, assisting in the deeper translocation of liquid film into the sections of the turbinate region (i.e., across T1, T2, and T3). 

When the CONV device was used in the more congested M1 model, there was notable upper-middle deposition in the T1 region. Additionally, the steeper head tilt led to some liquid film deposition in the middle meatus, which, under the influence of flow shear and gravity, moved and settled in the T2 region, as marked by a white arrow in the upper panels of Figure 9a. Moreover, as with the behavior at the 22.5° tilt, the liquid film that was initially deposited in the middle of T1 was pulled into T2, filling the narrow space between the septum and the inferior turbinate. However, at the 45° backward head tilt, the gravitational pull caused a more pronounced dragging of the liquid film toward the end of T2, leading to its accumulation/settlement there. In the M2 model, in which the CONV device is used, the wider passages allowed for more dispersed deposition within the upper-middle and lower halves of T1. This resulted in liquid film deposition on T1’s floor and its movement to the end of T2 along the floor. A slight deposition was also observed in the anterior middle meatus of T1, attributed to the steeper tilt.

The utilization of the MOD device brought forth similar deposition distributions in both the M1 and M2 models, as seen with the 22.5° backward head tilt, directly delivering liquid to the middle turbinate region and middle meatus. However, the steeper backward head tilt at 45° enhanced the gravitational force component along the middle meatus, aiding in deeper liquid film dragging while minimizing its translocation out of the middle meatus into lower turbinate regions along its path through the middle meatus. This condition led to a visibly greater liquid film deposition in the posterior and middle sections of both models (yellow arrows, Figure 9b, upper and lower panels), underscoring the large impact of device modification and head tilt on improving spray deposition.

**Figure 10:**
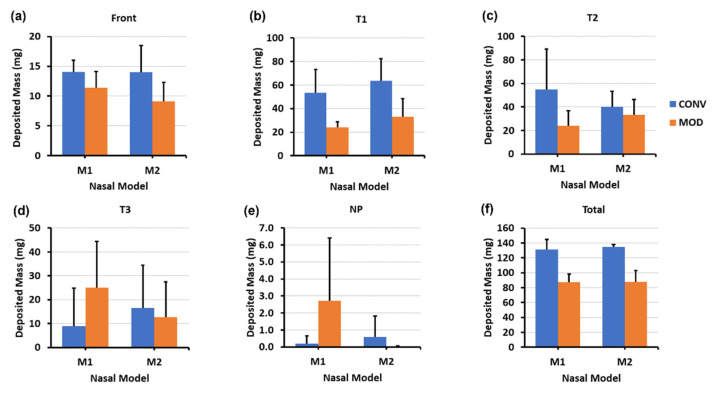
Deposition variation vs. nozzle type (retracting CONV and fixed MOD) in different regions of the nose with a 45° back tilt head position and a constant 16 L/min inhalation airflow for nasal models M1 and M2: (**a**) front nose, (**b**) T1, (**c**) T2, (**d**) T3, (**e**) nasopharynx (NP), and (**f**) total deposition.

The mass deposition distribution charts shown in Figure 10 indicate that, as with the 22.5° backward head tilt, the deposited mass within the various turbinate sections using CONV in both M1 and M2 was generally higher than that with MOD. Although the deposition with CONV was distributed across the lower half of the turbinate sections and along the nasal floor, MOD consistently led to uniform deposition throughout the middle meatus and the surrounding middle turbinate region, as well as at the lateral posterior turbinate region. Moreover, liquid sprays administered utilizing the steeper 45° head angle facilitated greater mass deposition in the posterior regions of the nasal cavity, namely within T3 and NP. Using CONV resulted in a deposited mass of 8.84 mg, whereas MOD led to a mass deposition of 25.06 mg within the M1 model in the T3 turbinate section. Deposition in T3 using CONV was across the floor, in contrast to when MOD was used, which led to more lateral deposition in the posterior turbinate region. Furthermore, utilizing a 45° backward head tilt assisted by inhalation flow led to lower deposition in the front nose section in both M1 and M2 nasal models using the MOD device compared to using CONV (i.e., 11.38 mg for MOD vs. 14.06 mg for CONV in M1, and 9.10 mg for MOD vs. 14.02 mg for CONV in M2). Note that some droplets escaped the nasal filtration with the carrier flow, exiting through the nasopharynx and being evacuated by the vacuum tube connected to it. This mass loss was likely attributed to the narrower plume angle of the MOD sprays, the gravitational force from a large backward head tilt angle (45°), and the flow-induced shear force.

#### 3.2.5. Violin Plots

**Figure 11:**
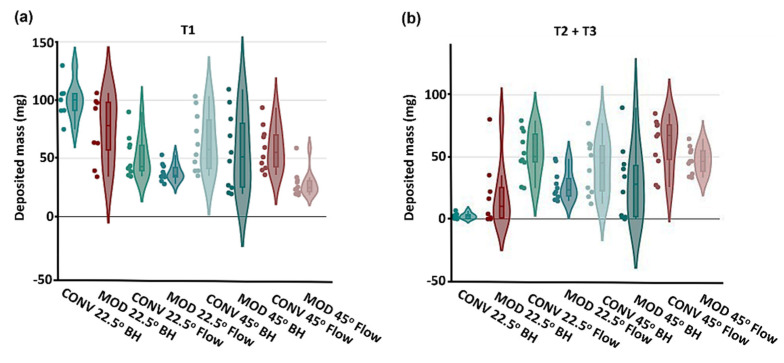
Violin plots of deposited mass in M1 and M2 combined vs. device, head angle, and inhalation scenario (breath-hold [BH] or constant inhalation [Flow]) in the various turbinate sections: (**a**) T1, and (**b**) combined T2 and T3.

Figure 11 presents the violin plots illustrating the combined deposition data statistics for both the M1 and M2 models across all study cases within the T1 and combined T2 and T3 sections. The corresponding average deposition and standard deviation (SD) are listed in Table 1. 

**Figure 12:**
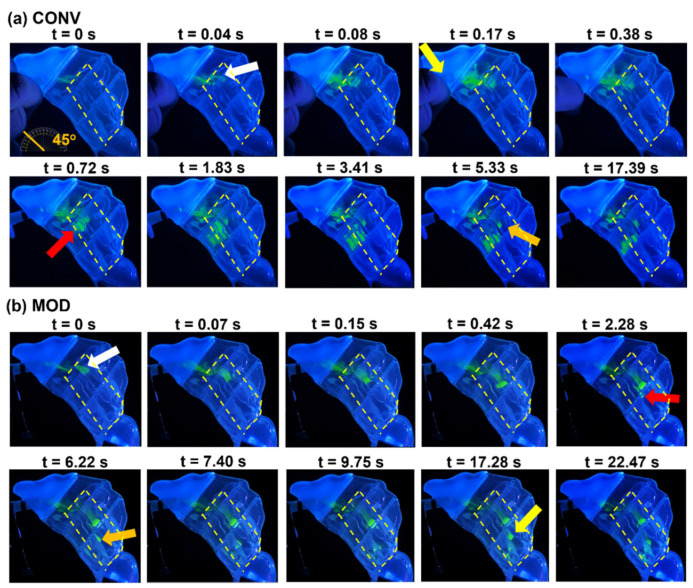
Time-series visualization of liquid film development and translocation in the M1 model at a 45° backward head tilt and constant inhalation vs. device type: (**a**) CONV, and (**b**) MOD. The target region is outlined in dashed yellow lines.

In T1, which represents the anterior section of the turbinate regions, the deposited mass was greater using CONV than MOD (Figure 11a and Table 1). Furthermore, with a set head angle and device, deposition in T1 generally decreased when spray administration was accompanied by inhalation flow compared to a breath-hold scenario. The impact of inhalation flow on enhancing delivery to the T2 and T3 sections is evident from the violin plots (Figure 11b). For the same head position and device, deposition in T2 + T3 sections was statistically greater (*p* < 0.05) with inhalation flow than with a breath-hold (i.e., no flow). Higher deposited masses in T2 + T3 are observed using CONV than MOD for all cases except the one with breath-hold and 22.5° head tilt (Table 1). However, it is noted that the mass distributions in the vertical direction (i.e., inferior, middle, and superior) are very different, which are shown graphically in Figure 3, Figure 5, Figure 7, Figure 9, and Figure 12, but have been missed in Figure 11 and Table 1.

### 3.3. Time-Series Visualization of M1 Spray Deposition

From a visualization perspective, the largest differences in deposition patterns between the CONV and MOD devices were observed with a 45° backward head orientation and a constant inhalation airflow rate of 16 L/min. Figure 7 depicts a time-series visualization of spray droplet deposition, liquid film development, and subsequent film translocation within the M1 model after dispensing a single dose of the 0.25% w/v MC aqueous spray solution from both the CONV (Figure 7a) and MOD (Figure 7b) devices.

#### 3.3.1. CONV

As depicted in Figure 12a, upon pressing down the finger-actuated applicator of the CONV device, which is integrated with the device’s tapered nozzle, spray dispensing from the nozzle tip was initiated at t = 0 s. In the early stage of spray plume development, spray droplets began to deposit in the middle T1 section within the region adjacent to the anterior middle and upper inferior turbinates, with a very slight film stream depositing in the anterior middle meatus (white arrow). At 0.08 s, as the actuator was pressed further, moving the nozzle tip further away from its initial depth within the nasal vestibule, the spray plume became larger, wider, and more forceful as a result of the heightened pressure behind the nozzle’s orifice. This resulted in a wider spread of liquid droplet and film deposition, both inferiorly and superiorly, within the middle T1 section, as well as additional liquid deposition in the anterior middle meatus extending slightly to the T2 section. By the 0.17 s mark, spray actuation had concluded, and the actuator together with the nozzle completed their full stroke length, retracting the nozzle’s tip a total distance of 5.5 mm (as illustrated by the schematic in Figure 1e) from its initial position within the nasal vestibule, as indicated by the yellow arrow in Figure 12a. After the completion of the spray application, segments of the liquid film that had developed within the T1 section and the anterior section of the middle meatus began to break up at t = 0.38 s due to the combined effects of flow shear and gravitational forces.

At 0.72 s, a portion of the liquid film that had split from the deposit in the middle T1 section translocated into the tight, narrow space between the outer wall of the inferior turbinate and the nasal septum lining. It initially covered the upper section of the anterior inferior turbinate wall and the adjacent septum lining within T2 (red arrow). As inhalation continued, by 1.83 s, gravity-assisted flow shear drew the film posteriorly, spreading it more uniformly across the outer wall of the inferior turbinate within the T2 section. After 17.39 s, this liquid film ceased to move and settled at the end of the inferior T2 section (orange arrow). Simultaneously, a liquid film that was initially deposited in the anterior middle meatus after spray application translocated deeper into the middle meatus due to the joint effects of gravity and flow shear, but predominantly because of the larger flow shear due to the dominant airflow through the middle meatus. Film translocation within the middle meatus ceased at the 5.33 s mark, where it finally settled within the middle meatus in the mid-T2 section (orange arrow).

#### 3.3.2. MOD

In contrast to the CONV device, the MOD’s design feature that fixes the nozzle in place within the nasal vestibule during spray application resulted in very different behaviors and deposition distributions within the M1 model. As shown in Figure 12b, right from the initial device actuation at t = 0 s, the narrower plume angle from the MOD device led to deeper initial spray penetration through T1, directly targeting the entrance to the middle meatus. This led to the deposition of liquid film in the anterior middle meatus, extending to the front of T2 (white arrow) and also within the middle T1 section, leading to the middle meatus. By 0.07 s, the continued spray application ensured consistent delivery to the middle meatus, resulting in the accumulation of liquid film in its anterior section.

After the completion of spray application at t = 0.15 s, the combined effects of flow shear, due to airflow through the middle meatus and the gravitational force along the slope of the middle meatus, facilitated the translocation of the accumulated liquid film deeper into the middle meatus. This process coated the meatus and led to the film settling and ceasing translocation upon reaching the end of the T2 section at t = 2.28 s. At this point, influenced by the vertical component of gravitational force, a segment of the accumulated liquid film detached and descended onto the outer surface of the inferior turbinate below (red arrow), gradually filling the narrow gap between the posterior outer wall of the inferior turbinate and the adjacent nasal septum lining (orange arrow). The film continued to translocate until it settled in the posterior region of the nasal cavity within T3 at the 9.75 s mark. By t = 17.28 s, destabilization of the liquid film deposited within the middle meatus at the end of the T2 section led to partial film breakup (yellow arrow) and further translocation of this film toward the posterior cavity within T3, ultimately merging with the settled liquid film in that section increasing the total deposition in the posterior region.

## 4. Discussion

This study systematically investigated the impact of eliminating nozzle retraction in a standard multi-dose nasal spray pump on the local delivery and deposition of liquid spray within the middle and posterior turbinate regions, comparing it against conventional nasal spray pumps characterized by retractable nozzles. The research considered multiple critical factors to thoroughly evaluate the deposition pattern differences between the conventional (CONV) and modified (MOD) devices. These factors included the administration angle, set at 30° relative to the nostril’s normal, and the insertion depth, maintained at approximately 10 mm into the nasal vestibule. These parameters were strategically selected to influence liquid deposition toward the desired middle and upper-middle regions of the nasal cavity beyond the restrictive nasal valve. Additionally, the study design incorporated varying inhalation scenarios—breath-hold and constant inhalation—to simulate different conditions of nasal spray application and assess their effect on spray distribution. The modified device’s design, aimed at eliminating nozzle retraction, was hypothesized to enhance the precision of medication targeting the middle and posterior regions of the nasal cavity. This adjustment is crucial for optimizing the therapeutic efficacy of nasally administered drugs, considering the anatomical and physiological complexities of nasal drug delivery.

### 4.1. Comparative Evaluation of Device Spray Characteristics

Modifications to the spray characteristics in the MOD device were primarily due to the integration of an elongated, flexible plastic tube. Despite employing the same force and speed during application, the average mass of liquid dispensed by the MOD device per dose application was found to be lower than that of the CONV device (143.2 mg vs. 160.3 mg, respectively). The addition of the tube introduced a longer path for the liquid to traverse, increasing flow resistance and affecting the dynamics of the spray. The flexibility and slack of the tube could also absorb some of the mechanical energy intended for spray generation, leading to a reduction in the volume of liquid dispensed. These modifications influenced aerosol dynamics, resulting in a slight increase in particle sizes (with the mean particle size for MOD at 101.704 μm vs. 90.456 μm for CONV, as shown in Figure 2a). The modifications could have also affected the spray plume’s exit velocity, contributing to a narrower plume angle without any observable alterations in the spray plume spread range between MOD and CONV.

Adjustments to future devices incorporating this feature might depend on the desired outcomes. For example, modifying the pump mechanism to counteract the increased flow resistance by extending its stroke length could enhance the pressure produced by the mechanism. This would help in maintaining particle size consistency with that of CONV and potentially optimizing it further based on specific formulation needs and targeted delivery areas. Such considerations underscore that modifications are contingent upon whether the observed spray characteristics align with the therapeutic goals.

### 4.2. Efficacy of the Modified Nasal Spray Pump (MOD) in Dosimetry

Overall, the CONV nasal pump delivered higher spray doses to both anterior turbinate (T1) and middle-posterior turbinate (T2 + T3) compared to the MOD nasal pump (Figure 11 and Table 1). Note that deposition in T1 cannot be absorbed effectively and is often considered as drug loss. A significantly lower T1 deposition using MOD is desirable. Considering the middle-posterior turbinate (T2 + T3), even though the delivered mass is higher using CONV than MOD, the CONV-delivered mass has been observed more frequently in the nasal floor, in contrast to the middle and superior regions of T2 and T3, as outlined by the yellow dashed lines in Figure 3, Figure 5, Figure 7 and Figure 9. Thus, the MOD device’s fixed-nozzle design led to more precise dosage control and targeted delivery, particularly beneficial in bypassing the nasal vestibule—a region characterized by squamous epithelium, which is less conducive to drug absorption. The narrowing of the spray plume angle with MOD, as opposed to CONV, facilitated this by enabling deposition deeper within the nasal cavity, directly targeting the middle meatus in vitro. This finding aligns with existing literature, where narrower plume angles have been associated with enhanced posterior deposition. Cheng et al. discovered that increased deposition in the anterior nasal region is associated with larger droplet sizes and broader spray angles [21]. Similarly, Foo et al. observed that sprays characterized by narrower plume angles were more likely to deposit in the posterior nasal area, showing little influence from the size of the droplets or their viscosity [41].

In scenarios where no inhalation flow was applied (i.e., breath-hold), MOD demonstrated an enhanced ability to deposit medication more effectively within the middle and upper-middle sections of the T1 turbinate area and the anterior middle meatus, as evidenced by the comparative analysis of liquid deposition patterns in Figure 3 and Figure 5. This outcome highlights the device’s effectiveness in overcoming the conventional limitations associated with nozzle retraction. Compared to CONV, MOD prevented the tendency for deposition along the nasal cavity floor under both experimental conditions of breath-hold and head tilts (22.5° and 45° backward)—a region suboptimal for drug absorption due to its reduced vascularization and limited surface area exposure.

Under conditions simulating a 22.5° and 45° backward head tilt coupled with constant inhalation at 16 L/min, the fixed-nozzle design of MOD distinctly improved liquid delivery to the middle turbinate region, including the middle meatus, and notably to the posterior turbinate regions. These turbinate regions, rich in vascular networks, are more conducive to rapid absorption, representing key target areas for efficacious drug delivery. The posterior two-thirds of the nose and nasopharynx, areas abundant in ACE2 receptors, are pivotal for effective mucosal immunization [9,10,11]. This emphasizes the critical importance of targeted delivery to the posterior nasal regions for achieving the desired immunological response. Additionally, accurately targeting the middle meatus is recognized as an essential approach in treating conditions such as nasal polyposis and chronic rhinosinusitis [13,25,26]. This strategic focus underscores the significance of precision in nasal spray application to achieve optimal therapeutic outcomes in managing these ailments. Figure 7, Figure 9 and Figure 12 highlight these deposition enhancements, showcasing MOD’s superior performance in achieving uniform deposition across the middle meatus and adjacent areas as well as high deposition in the posterior turbinate regions. This outcome underscores the device’s proficiency in overcoming the conventional limitations associated with nozzle retraction, which, under a breath-hold and both head angles as per the experimental design (22.5° and 45° backward tilts), tended to favor deposition along the nasal cavity floor—a region less optimal for drug absorption due to its lower vascularization and limited surface area exposure.

Conversely, using the conventional device (CONV) under inhalation flow in both head positions, deposition in the M2 model was predominantly along the floor, not extending beyond T2. In the M1 model, however, liquid deposition also spanned the lateral inferior turbinate across, which still did not extend beyond T2. This highlights a less targeted approach with CONV, which might not fully utilize the therapeutic potential of nasal spray medications in critical regions. Furthermore, under constant inhalation flow and a 45° backward head tilt, the front nose sections of M1 and M2 exhibited marked differences in deposition between MOD and CONV, underscoring the modified device’s potential to reduce drug wastage and improve treatment outcomes.

### 4.3. Clinical Significance and Implications of the Modified Nasal Spray Pump

The Modified (MOD) nasal spray pump introduces enhanced precision and targeted deposition capabilities, pivotal for achieving clinical efficacy. By fixing the nozzle’s position and eliminating the retraction characteristic of conventional nasal spray pumps (CONV), the MOD device demonstrates a pioneering approach to overcome common limitations in nasal spray application, particularly in delivering medication to the middle and posterior regions of the nasal cavity. The clinical implications of this innovation are profound. Utilizing a constant inhalation flow of 16 L/min and a 45° backward head tilt, as outlined in this study’s protocol, the MOD device’s capacity to deliver medication accurately to the middle meatus and posterior turbinate regions—areas densely vascularized—promises to notably enhance the absorption and efficacy of treatments. This is especially relevant for conditions where rapid onset of action is critical, such as acute migraine relief, or for conditions requiring targeted delivery, such as chronic rhinosinusitis or nasal polyposis management. The observed trend toward enhanced delivery to these regions, as evidenced by uniform deposition and increased reach to the posterior turbinate areas, underscores the MOD device’s potential to improve therapeutic outcomes.

Moreover, adhering to the same protocol, the trend toward reduction in medication waste, as indicated by decreased deposition in the less absorptive anterior nasal vestibule, aligns with a more efficient and cost-effective use of medications. This aspect is particularly meaningful in the context of costly drugs or vaccines, where maximizing the bioavailability of each dose is crucial [42,43]. The significance of the MOD device also extends to the realm of vaccine administration, particularly for vaccines targeting respiratory viruses like influenza or SARS-CoV-2 [44,45,46]. The potential to deliver vaccines effectively to the posterior two-thirds of the nose and nasopharynx, regions rich in ACE2 receptors, could enhance mucosal immunization and contribute to more effective protection against these pathogens [47,48].

### 4.4. Study Limitations

While this study presents critical insights into the improved performance of the MOD device over conventional nasal spray pumps, it is not without limitations. The inability to quantify the exact distribution of deposition across each turbinate region section highlights the need for more refined models or methodologies that can facilitate precise measurement of regional deposition mass. Also, though the anatomically accurate nasal models used were essential for evaluating nasal spray deposition, they represent a limited range of nasal geometries. Including a wider variety of patient-specific nasal models would offer a better understanding of structural variability on spray distribution, enriching evaluations of nasal spray effectiveness across diverse populations.

Additionally, the SLA resin used to print the nasal models does not mimic the properties of the actual nasal airway tissue, which includes squamous epithelial tissue at the front and nasal mucosa in the turbinate area [49,50]. An in-depth comparison of the interactions between liquids and the nasal mucosa versus those with the resin surface is necessary to gauge how closely the models replicate real nasal cavity conditions and what this means for the results of the study [51,52]. Of note, the SLA resin is non-biomimetic and does not allow for the evaluation of mucociliary clearance and cilia beating [53,54]. These factors can be important in dose translocation and thus may affect the extrapolation of the results to in vivo conditions. Furthermore, the model’s sections were connected using tongue-and-groove joints, which were not completely watertight. As a result, the MC solution occasionally leaked through these connections by capillary action, causing slight inaccuracies in measuring deposited amounts. While these variances were considered minor, they warrant recognition. Furthermore, the choice of a single formulation (0.25% MC aqueous solution) limits the generalizability of the study’s findings across different drug formulations with varying viscosities and properties [55,56]. Considering the nasal device characterization, the spray exit velocities were not considered in this study but could be quantified using either phase Doppler anemometry (PDA) or high-speed particle image velocimetry (PIV) [57,58,59].

## 5. Conclusions

This study presents a comparative analysis between a modified standard multi-dose nasal spray pump (MOD) and a conventional nasal spray pump (CONV). The MOD device, characterized by a fixed nozzle, was designed to address the challenge of nozzle retraction inherent in CONV devices, aiming for improved intranasal drug delivery to the middle and posterior regions of the nasal cavity. The study demonstrated that the MOD device’s fixed-nozzle design leads to a narrower spray plume, facilitating more targeted and efficient spray delivery. This is particularly evident in its ability to direct medication to the targeted middle-upper nasal regions as opposed to the nasal floor, which is less responsive to medication. By contrast, a large portion of CONV-delivered doses are deposited on the nasal floor, rendering them less effective even though the total mass in T2 and T3 is larger than that with MOD. Enhanced delivery to the target (middle-upper regions of T2 and T3) using MOD was observed. Such delivery enhancements are crucial for maximizing therapeutic outcomes in treating nasal and sinus conditions.

Moreover, the study’s findings suggest a trend toward reduction in drug wastage in the front nose and nasal floor with the MOD device, particularly under specified inhalation and head tilt conditions. This outcome suggests the MOD device’s potential to enhance the efficiency of medication use, particularly for high-cost or limited-availability drugs. While the precision of the MOD spray pump is notable, higher drug losses to the pharynx have also been observed, particularly with a 45° backward head tilt position and inhalation flows. The conclusions drawn from this study are preliminary. However, they suggest the MOD device’s promising contributions to intranasal drug delivery technologies, pending further validation with in vivo experiments to better assess the prediction potential for human diseases.

## Figures and Tables

**Table 1:** Comparison of the averaged and variance of deposition using CONV and MOD.

	(mg)	CONV 22.5° BH *	MOD 22.5° BH	CONV 22.5° Flow	MOD 22.5° Flow	CONV 45° BH	MOD 45° BH	CONV 45° Flow	MOD 45° Flow
T1	Avg	99.80	74.51	51.43	37.74	63.65	55.52	58.59	28.52
	SD	17.00	28.22	17.77	7.43	25.22	33.46	18.98	11.79
T2 + T3	Avg	2.47	19.89	52.88	27.26	42.2	29.02	60.07	47.38
	SD	2.37	27.49	17.79	12.23	22.05	29.41	21.26	10.74

* BH: breath holding.

## Data Availability

The data presented in this study are available on request from the corresponding author.
